# Use of the Anatomage Virtual Table in Medical Education and as a Diagnostic Tool: An Integrative Review

**DOI:** 10.7759/cureus.35981

**Published:** 2023-03-10

**Authors:** Mohamed Atif A Said Ahmed

**Affiliations:** 1 Department of Basic Medical Sciences, College of Medicine, Shaqra University, Shaqra, SAU

**Keywords:** medical curriculum, anatomy, diagnostic tool, medical program, residency program, virtual table, anatomage

## Abstract

Studies on human anatomy mainly depend on cadaver dissection. New technology devices have progressed to improve anatomy teaching, such as the 3D Anatomage virtual dissection table. There is a shortage and deficiency in information about the uses and benefits of the Anatomage table. The aim of this article was to review and assess the current evidence about the advantages of the Anatomage virtual table in medical education and curricula of medical courses, and its utility in diagnosis.

The current study is a comprehensive systematic review. A search was conducted on online medical and scientific databases. Twenty-nine articles relevant to the content of the current research topic were selected. Based on this review, the use of the Anatomage table is valuable for anatomy learning outcomes, and most of the research supported it as an important anatomy tool in addition to cadaveric dissection. The Anatomage table in association with human dissection can improve knowledge retention of anatomy. It is an important tool for understanding organ variation. Anatomage is now considered an important tool for the educational training programs of medical students and residents and for disease diagnosis and prognosis. Anatomage can make the curriculum more interesting and valuable. Utilizing the Anatomage table can help medical and paramedical students and residents by assisting them to understand anatomy in a better way. It will also improve radiological knowledge and facilitate pre-planning for surgeries. Finally, it has a crucial role during exceptional circumstances such as pandemics.

## Introduction and background

Human anatomy is fundamental in learning the basic medical sciences and serves as a gate for all clinical courses. It has traditionally been taught via cadaveric dissection and didactic lectures [1]. Cadaveric dissection was the cornerstone of the medical curricula [2]. Anatomical knowledge is critical for the accurate diagnosis and treatment of many diseases [3].

There is an ongoing discussion about the importance of cadaveric dissection against the cost, ethical rules, shortage of cadavers, and facility management of the dissection lab [4]. Now, there is an increased shift in attitude toward the need of using cadaveric dissection in the curricula of medical and paramedical colleges. The dissection procedures and identification of fine anatomical structures will affect negatively the time consumed in the curricula. The quality of dissection became stressful for the students. The number of students in the class is increasing and the availability of cadavers may be inadequate for the student’s requirements.

The increase in technological resources for medical education and the need for knowledge retention requires staff to find more creative learning methods [5]. Many studies recommended using information technology in teaching, which can positively affect students' performance during exams [6]. New paramedical students have been interested in employing technology for learning [7].

Recently, most medical programs have moved from teacher-centered learning to student-centered learning. The increase in information technology has an essential role in the field of medical education. Changes include utilizing the new technology virtual dissection table has constructive feedback from the students about spatial knowledge of human anatomy [8]. The Anatomage table is a useful tool to allow the simulation of human dissection to be so close to reality. It is a touch-based interactive anatomy used in anatomy education that improves the student's perception of anatomical knowledge and anatomy learning to promote students’ thinking; this will consequently lead to improve retention of knowledge [9,10].

The virtual anatomy table (Anatomage) life-size was designed by a corporation in the USA in association with the Anatomy department at the Medical College of Stanford University. It was developed using a series of multidimensional photos of different parts of the human body. The photos are reconstructed to represent the full body by using a special software program. The student can dissect the reconstructed human body and remove the superficial layers to illustrate the deep layers and organs. Many sections in multiple axes can be done to demonstrate the anatomical relations of variable structures of the human body. It is possible to change the gender in the Anatomage table. The Anatomage virtual table has the facility of radiological imaging (CT/MRI) to support the integration of anatomy and radiology in the curricula. Anatomage has been used recently in maxillofacial and general surgery courses [11]. It has the advantage of being capable of reuse multiple times [12,13]. The Anatomage virtual table does not need any ethical approval compared to cadaveric dissections. It also has numerous advantages and can be a valuable addition to anatomy learning.

There is a shortage and deficiencies in information about the Anatomage table, its facilities, and its benefits.

The present article aimed to 1) Review the benefits of using the Anatomage table in the field of medical education and elaborate on its uses for medical students, residents, and paramedical students. 2) Understand the basics of using the Anatomage tool to acquire competent clinical skills. 3) Highlight the use of Anatomage that might help in diagnosis and its uses in radiology and surgery.

## Review

Search methodology

The current study was a comprehensive systematic review that was carried out from April 2022 to October 2022. This article review was conducted by using databases on the following online sites: National Library of Medicine, PubMed, Embase, SCOPUS, Wiley Online Library, Google Scholar, Ovid MEDLINE, ERIC, and Cochrane. The databases were searched for articles published in the English language using the keywords "Anatomage ", "Virtual anatomy", "Virtual dissection table", "Residency training program", "Undergraduate training program", and "diagnostic virtual tool". We searched for articles with the purpose of exploring the outcomes of the use of Anatomage tables in conjunction with cadaver anatomy education. Literature on the use of the Anatomage table in undergraduate and postgraduate medical student programs and paramedical programs was also reviewed. We are following Preferred Reporting Items for Systematic Reviews and Meta-Analyses (PRISMA) guidelines.

Research matching the keywords was revised. We used a Microsoft Excel sheet (Microsoft Corporation, Redmond, WA) to complete the selected data. The present study is descriptive, so no statistical analysis was used.

Eligibility criteria

All research papers that included Anatomage were used (1) for measuring the student learning outcome, (2) for diagnosis, (3) for radiology, and (4) for pre-planning surgeries. Duplicate studies were deleted. Reference lists from the selected articles were also checked to increase the validity of the search. The articles included case reports, letters to the editor, expert opinions, short messages, textbook chapters, and animal studies. Only articles published within the last twenty years were included.

Results

We selected the research papers that suitably described the title of our review. The abstracts of the selected papers were reviewed before going to the details of the research paper.

**Figure 1:**
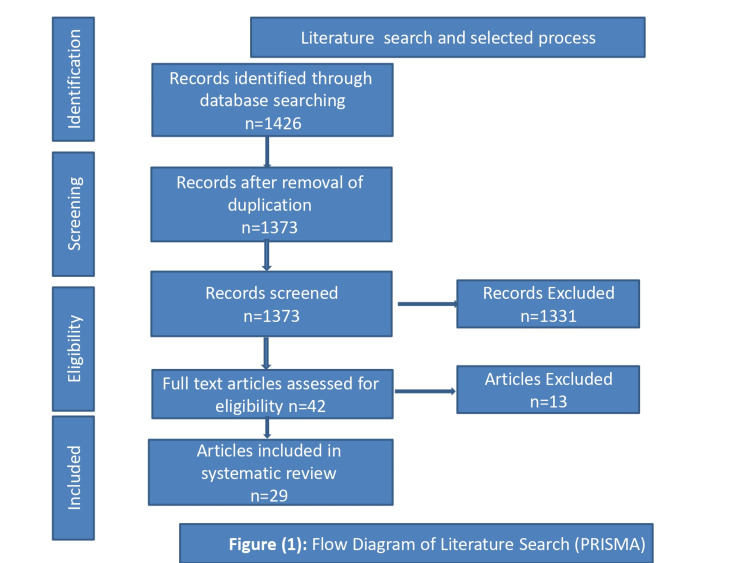
PRISMA chart for literature search PRISMA: Preferred Reporting Items for Systematic Reviews and Meta-Analyses

The flow diagram of the literature search (PRISMA) is represented in Figure 1. The search resulted in 1426 research papers. Duplicated papers were removed, which yielded 55 research papers. Finally, 29 research papers were selected after deleting the irrelevant papers. The study characteristics are listed in Table 1.

**Table 1:** Systematic review of studies and results findings

Study Title	Year Published	Authors	Study Design	Test	Sample Characteristics	Results
Morphometric analysis of bone resection in anterior petrosectomies	2016	Ahmed O, Walther J, Theriot K, Manuel M, Guthikonda B [14].	A cadaveric study	Measure the bone resection and the improved viewing angle.	14 anterior petrosectomies on eight cadaveric heads. Pre and post-dissection CTs were obtained using the Anatomage table.	Anatomage is useful in preoperative information for approach and gaining the viewing angle after bony removal.
Analysis of immediate student outcomes following a change in gross anatomy laboratory teaching methodology.	2018	Afsharpour S, Gonsalves A, Hosek R, Partin E [15].	Prospective study	Students’ scores in an objective structural practical exam and final exam.	352 students used cadaveric dissection, 350 students used the plastinated model and 393 students used the Anatomage virtual table.	Students who used the Anatomage tool showed significant improvement in their scores on the objective structural practical exam.
Medical students' feedback on applying the virtual dissection table (Anatomage) in learning anatomy: a cross-sectional descriptive study.	2021	Alasmari WA [16].	A cross-sectional descriptive study.	An electronic questionnaire	78 medical students.	The students preferred using Anatomage as it improves active learning. About 90% of students voted for the benefits of Anatomage in understanding anatomical relationships. Approximately 72% of the students indicated that Anatomage was useful for enabling their understanding of anatomy.
A comparative study of learning with “Anatomage” virtual dissection table versus traditional dissection method in neuroanatomy	2017	Anand MK, Singel T [17].	Prospective study	A questionnaire was done before and after the test.	Anatomage virtual dissection tool versus traditional dissection (n=122 students).	Most of the students believed that the Anatomage table improved classroom experience but no significant difference between the two groups
Evaluating the Anatomage table compared to cadaveric dissection as a learning modality for gross anatomy.	2019	Baratz G, Wilson-Delfosse AL, Singelyn BM, Allan KC, Rieth GE, Ratnaparkhi R, Jenks BP, Carlton C, Freeman BK, Wish-Baratz S [18].	A crossover study	Survey before and after each lab and formative assessment test after each lab.	Four groups were used in this study. Each group consisted of four students. Groups were randomly selected to study on the Anatomage table and not on the cadaver lab or vice versa.	Students were more excited by using Anatomage and perceived a greater degree of learning. Students group used Anatomage showed significantly higher grades on exams.
A study on student perception of virtual dissection table (Anatomage) at GSL Medical College, Rajahmundry.	2018	Bharati AS, N SK, Rani VS [19].	Cross-sectional study	Questionnaire	150 students	More than 90% preferred the Anatomage dissection table to cadaveric dissection and textbooks.
Evaluation of the effectiveness of digital technologies during anatomy learning in nursing school.	2020	Bianchi S, Bernardi S, Perilli E, Cipollone C, Di Biasi J, Macchiarelli G [20].	Prospective- cohort study	Questionnaire	130 students (Anatomage use vs no Anatomage use).	Anatomage use has a significant improvement in exam scores.
Students’ perceptions and attitudes after exposure to three different instructional strategies in applied anatomy.	2021	Bin Abdulrahman KA, Jumaa MI, Hanafy SM, Elkordy EA, Arafa MA, Ahmad T, Rasheed S [21].	Cross-sectional study	The objective structured practical examination was given to all students,	First-year medical students were distributed into three groups. Group A learned with the "Anatomage" and Group B learned about plastinated specimens. Group C was learning about plastinated specimens and the "Anatomage" table.	Medical students expressed a higher attitude towards using Anatomage and plastinated specimens compared to the Anatomage table alone or plastinated specimens for teaching.
The additional role of virtual to traditional dissection in teaching anatomy:	2021	Boscolo-Berto R, Tortorella C, Porzionato A, Stecco C, Picardi EE, Macchi V, et al [22].	A randomized controlled trial.	A survey was done before and after the test.	Ten students using Anatomage vs 13 students using textbooks.	Significant improvement in learning outcomes was shown in the group of students using the Anatomage.
Medical student perception of Anatomage–A 3D interactive anatomy dissection table	2015	Brown J, Stonelake S, Anderson W, Abdulla M, Toms C, Farfus A, et al [23].	Cross-sectional study	Questionnaire	511 medical students.	More than 75% of the students said that Anatomage made learning more engaging. More than 60% of the students said that it improved 3-D anatomy understanding.
The Anatomage table and the placement of a titanium mesh for the management of orbital floor fractures.	2018	Brucoli M, Boccafoschi F, Boffano P, Broccardo E, Benech A [11].	Prospective observational study.	Questionnaire	10 medical resident students (CT images vs Anatomage virtual table).	87% replied that the Anatomage virtual table provides better clarity in comparison to the CT scan.
The utilization of the Anatomage virtual dissection table in the education of imaging science students.	2015	Custer TM, Michael K [7].	Qualitative Case study	Revision of activities inside the classroom, small group sessions, and interviews.	Seventeen students from the department of medical imaging science were selected.	The majority of the students replied positively to the utilization of the Anatomage virtual table in their education.
Integrated virtual and cadaveric dissection laboratories enhance first-year medical students' anatomy experience.	2019	Darras KE, Spouge R, Hatala R, Nicolaou S, Hu J, Worthington A, et al [24].	A pilot study.	Evidence-informed survey.	292 medical students.	More than 78% said that the virtual dissection table enhanced their understanding of cadaveric anatomy.
Use of Anatomage tables in a large first-year core unit.	2013	Fyfe G, Fyfe S, Dye D, Crabb H [25].	Cross-sectional study.	Surveys	Three hundred twenty-six first-year medical students and 22 tutors shared in this survey.	Medical students mentioned that Anatomage was a valuable tool for demonstrating the different anatomical structures. Tutors were disappointed due to the technical excitement.
The Anatomage table: Differences in student ratings between initial implementation and established use.	2018	Fyfe S, Fyfe G, Dye D, Radley-Crabb H [26].	Cohort study	Questionnaire	Six hundred fifty-seven students share in this study.	Anatomage exhibited a good 3-D demonstration of virtual anatomy and supported the learning outcome. The introduction of Anatomage into the new curriculum needs good curriculum reconstruction.
Anatomy observational outreach: a multimodal activity to enhance anatomical education in undergraduate students.	2019	Gonzalez-Sola M, Hyder A, Rosario M [27].	Cross-sectional study	Orientation class, cadaveric dissection, Anatomage virtual table, and textbooks. A questionnaire was done before and after the test.	One hundred students.	More than 95% of students’ anatomical knowledge increased. More than 80% of students developed more interest in graduate degrees. Less than 20% believed that cadaveric dissection was the strongest educational learning method.
Virtual dissection: Using active learning with the Anatomage table to enhance student learning.	2017	Gross M, Masters C [28].	Observational prospective study.	Student feedback	One hundred twenty-three students.	Generally good reply and acceptance of using Anatomage table.
An efficient approach based on 3D reconstruction of CT scan to improve the management and monitoring of COVID-19 patients.	2020	Hasni M, Farahat Z, Abdeljelil A, Marzouki K, Aoudad M, Tlemsani Z, et al [29].	A prospective analysis.	Computer processing to obtain a 3D visualization.	CT images of 185 COVID-19 patients by using the Anatomage table.	3D reconstruction has a significant role in the diagnosis and management of COVID-19 patients.
Health professions student perceptions of the Anatomage virtual dissection table and digital technology.	2020	Kar R, Skaggs S, Wang H, Nation H, Sakaguchi AY [30].	Observational prospective study.	A questionnaire was done before and after the course.	Forty-four occupational therapy doctorate and 48 physician assistants.	More than 65% believed that virtual tables facilitate the understanding of anatomical structures due to their high resolution.
3D dissection tools in Anatomage supported interactive human anatomy teaching and learning.	2019	Kažoka D, Pilmane M [10].	Cross-sectional study	Students' feedback	200 students Anatomage vs classroom teaching.	The Anatomage table significantly improved the visualization and memorization of different anatomical structures.
Perceptions between medicine students on the use of Anatomage and other practice methods for anatomy teaching.	2020	Martín JG, Fernández LM, Arce AS de [31].	Cross-sectional study	Survey	32 students	47% found cadaveric dissection more useful while 6% found the Anatomage table very useful.
Cadaver-specific CT scans visualized at the dissection table combined with virtual dissection tables improve learning performance in general gross anatomy.	2017	Paech D, Giesel FL, Unterhinninghofen R, Schlemmer HP, Kuner T, Doll S [32].	Cohort study	Questionnaire	90 students used radiology seminars while 98 students used Anatomage vs 50 students used both radiology seminars and Anatomage.	Radiology seminars and Anatomage optimized the anatomical knowledge.
Anatomage virtual dissection table: A supplemental learning aid for human anatomy education during an undergraduate outreach activity.	2019	Rosario M, Gonzalez-Sola M, Hyder A, Medley A, Weber M [33].	Cohort study	Questionnaire	100 students Cadaveric dissection vs Anatomage virtual table.	More than 80% considered undergraduate learning of anatomy and physiology without Anatomage is lacking.
Digital anatomy table in the teaching-learning process of the temporomandibular joint anatomy.	2022	da Silveira CR, Miamoto Dias PE, Oenning AC, de Brito Junior RB, Turssi CP, Oliveira LB [34].	Cross-sectional study	Questionnaire	Forty-one students share in this study. Traditional lab group vs Anatomage group.	Median scores significantly increased with the Anatomage group showing higher grades and a good understanding of anatomy compared to the traditional lab group.
Virtual dissection table in diagnosis and classification of Le Fort fractures: A retrospective study of feasibility.	2020	Stecco A, Boccafoschi F, Falaschi Z, Mazzucca G, Carisio A, Bor S, et al [35].	Retrospective study.	Evaluation	10 trauma patients.	Inter-observer agreement > 90%.
Teaching gross anatomy: Anatomage table as an innovative line of attack.	2020	Tenaw B [36].	Cross-sectional survey	Questionnaire	89 students.	More than 80% agreed that Anatomage is a valuable additional tool for learning human anatomy.
Virtual dissection: Alternative to cadaveric dissection for a pregnant nurse anesthesia student.	2020	Washmuth NB, Cahoon T, Tuggle K, Hunsinger RN [37].	Case study	Theory and objective structural practical exams.	25 students shared in this study. One group used cadaveric dissection vs virtual Anatomage table group.	The Anatomage table added more advantages over cadaveric dissection with similar outcomes.
An innovative technique to promote understanding of anatomy for nurse practitioner students.	2019	Whited TM, DeClerk L, Berber A, Phelan KD [38].	Cohort study	Pre and post-tests on anatomy.	17 nurse practical students.	Clinically significant improvement in all areas except in the head and neck sections.
The impact of COVID-19 on plastic surgery residency training.	2020	Zingaretti N, Negrini FC, Tel A, Tresoldi MM, Bresadola V, Parodi PC [39].	Cross-sectional study.	Questionnaire feedback.	One hundred fifteen plastic surgery physicians.	Most of the plastic surgery physician’s feedback supported the idea that Anatomage is saving time in theoretical learning and in surgical procedures and it will help in resident programs.

The current review tries to highlight the uses of the Anatomage table in medical education and as a diagnostic tool.

Anatomage utility in anatomy for medical students

Cadaver dissection had proven to be the ideal method of learning anatomy. However, it has many restrictions. Dissection of cadavers is a stressful experience that causes many symptoms among medical students such as shortness of breath, increased heart rate, nausea, and vomiting. These limitations can be solved by using the Anatomage virtual table. We need to understand the 3D Anatomage virtual table and introduce it into medical education and curricula. The incorporation of Anatomage is inspiring ways in anatomy education [40]. It has many benefits compared to traditional teaching. Anatomage has different facilities as multi-axis sections of cadavers, quizzes, and CT/ MRI data. Accuracy in studying different human body parts and making different sections are beneficial to understand human anatomy. Applying anatomical knowledge in clinical practice by using the facilities of CT/MRI imaging that are present in the Anatomage table. It saves time in embalming or dissection. It is economical considering its unlimited use, and its software can be updated regularly. It shows real lifesize full-body gross. Compared to cadavers, no ethical approval is required with freedom of action.

The Anatomage virtual dissection table can be used in accordance with cadavers or even as a single tool for anatomy education. It offers learning anatomy and illustrates the fine details of different anatomical structures. Various layers of cadavers can be removed or sliced to enhance the student's interest. Students can also isolate a single organ by deleting the related structures. Anatomage can be also used for quizzes and commentary.

Afsharpour et al. reported that there is a marked improvement in the scores of practical exams among the students using Anatomage with no changes noticed in the scores of theory exams [15]. Alasmari noted that more than 80% of the participating students favored Anatomage as an extra tool in addition to cadavers to gain a good understanding of human anatomy [16]. The application of this new technology tool supports anatomy education. Brown et al. reported that most of the students voted positively regarding the involvement of Anatomage tables as a good learning tool [23]. Bharati et al. mentioned that there is an increased preference for Anatomage over textbooks [19]. In the neuroanatomy course, Anatomage proved to be a superior learning tool compared to cadaveric dissection. Anand et al. noted that close to 80% of students believed that the Anatomage tool enhanced the classroom learning process [17]. Brown et al. reported that Anatomage made medical students more actively involved in the learning process [23]. In another study, Anatomage appeared equal to the process of cadaver dissection [18].

A cross-sectional study on first-year medical students of Imam Mohammad Ibn Saud Islamic University reported that the Anatomage with plastinated specimens is more effective compared to the cadaveric dissection in anatomy learning, as the students can understand the anatomical location and the surrounding area when using Anatomage as compared to cadaver dissection [21]. da Silveira et al. noticed higher median scores of teaching strategy with Anatomage in students' perception of temporomandibular joint anatomy [34]. Similarly, Kažoka et al. noticed that the Anatomage table improved visualization and memorization of the different anatomical organs [10]. Anatomage is an excellent tool to learn human anatomy. Alasmari reported that the majority of medical students agreed that the Anatomage table improved their ability to study and learn the different anatomical structures [16]. She also reported that most of the students believed that visualization of the digital human body is easy in Anatomage due to its ability to rotate the images in a multi-axis [16]. In addition, many students benefitted from the integration of radiological imaging in Anatomage with digital cadaveric dissection as a preparatory step for the clinical stage.

Fyfe et al. mentioned that students get benefitted from the Anatomage for organ visualization, structure, and their relationship. However, students were not completely satisfied with the graphics [26]. The staff in the same study were disappointed with technical errors and the cost of the Anatomage table but they are in agreement with the usage of Anatomage. Tenaw and his team in their research reported that more than 80% of medical students were satisfied with using the Anatomage virtual table [36]. Paech et al. mentioned that the grades of student groups using cadaveric dissection and CT scans in virtual dissection tables together were significantly better than others using cadaveric dissection only.

Most of the previous studies reported that the students had positive feedback regarding Anatomage as an additional tool for teaching and learning human anatomy [10].

Knowledge of congenital anomalies and variations is important for medical student education. During cadaveric dissection, some congenital anomalies and variations are detectable and they can be loaded in the Anatomage table. The cadaver of one Asian lady who suffered from gastric carcinoma with respiratory failure was loaded in the Anatomage table. This case shows an alteration of the suprascapular veins in their numbers and courses [41]. Another cadaver of a Caucasian man shows abnormal variation in the anatomical course of the mandibular nerve was uploaded [42].

Few studies mentioned that students preferred traditional cadaveric dissection to Anatomage [31]. Purchase orders and the need for Anatomage has increased over the year, which reflects the time for integrating the new technology tool in medical education [26].

Some authors reported the disadvantages of the Anatomage table where congenital anomalies and variations in the human body cannot be identified. No pediatric anatomy is provided. The touch feeling of anatomical structures cannot be acquired, and the tissue color is different from the real cadaver [19,40]. It is costly and will need to be updated regularly.

Anatomage utility for paramedical students

Custer and Michael mentioned that students in the imaging science department benefit from using Anatomage and the majority of students gave good feedback about Anatomage, as it had a positive effect on their learning ability of anatomy [7]. The Anatomage table was successfully used for nursing students at Mac Ewan University [43]. It can also be used as an effective tool in forensic odontology education and training [44].

Anatomage utility in curriculum

Human anatomy is considered a cornerstone of the medical curriculum. Dissection has been marginalized in medical curricula due to the deficiency of cadavers, shortening of curricular time, cadaveric costs, and maintenance of anatomy dissection labs [3,45]. Introduction of Anatomage into a medical, paramedical, and residency training curriculum needs to concentrate on its importance and advantages and explore the affiliation with students’ requirements. Anatomage enhanced active learning by allowing the students to answer questions and discuss and share thoughts on anatomical knowledge, hence improving communication skills [28]. Anatomage facilitates the development of skills for self-dependent study, scientific reasoning, and interpersonal skills [46]. Incorporation of Anatomage into the medical curriculum is beneficial for students [30]. Anatomage also has a positive effect on medical education and curriculum. The overwhelming evidence suggests that Anatomage would be a very important tool in residency, medical student, and paramedical students' training programs.

Anatomage utility in residency training

In fields other than undergraduate medical students, the Anatomage provides an excellent opportunity for residents to update their knowledge and anatomy learning. Some researchers reported an improvement in the learning outcome of residents when they combined cadaveric dissection with Anaotmage [22].

Anatomage represents an assistant value for surgeons. It could help junior surgeons practice on a virtual cadaver. They can also make a valuable discussion about surgical cases and can make the preoperational plan. Stecco and his colleagues reported that more than 90% of participants favored Anatomage over radiology in a study to estimate the uses of Anatomage as a diagnostic tool to perform maxillofacial CT for patients with Le Fort fractures [35]. It can also be used in round meetings to facilitate the learning outcome and for the application of any novel surgical techniques. Brucoli et al. noted that 87% of the participants of maxillofacial surgery residents and junior surgeons believed that Anatomage is an excellent diagnostic tool in pre-operative surgical planning [11]. The Anatomage table is important in maxillofacial surgery for the diagnosis and evaluation of head and neck tumors. The lesions can be identified by residents. They can study all neck tumors in multiple axes to determine their size, grade, and strategy for the preoperational plan [47].

Anatomage utility in radiology

Anatomage also has an important role in radiology. There is a possibility to see Anatomage radiological images close to cadaveric projections [18]. Anatomage may be used in research and multi-disciplinary meetings to explain the findings. Radiologists can make interpretations of the images to demonstrate any pathological conditions. Radiological images are preinstalled onto the Anatomage, including CT and MRI. Medical students and radiologists can integrate CT/MRI radiological images with 3D cadaveric views. Anatomage helps the students understand radiological images of human skeletons in their normal size with fine details and to understand the anatomical position and relation of each bone [48].

Anatomage utility in diagnosis

An accurate diagnosis of any disease requires a deep knowledge of human anatomy. Anatomage is an excellent diagnostic tool. Radiological images of any patient can be uploaded and reconstructed by using the software program. It will be possible to study any case of different diseases on Anatomage.

In cases of pelvic and intra-articular fractures, fractures can be diagnosed using Anatomage by the radiologist. CT/MRI images of the patients can be uploaded for accurate diagnosis [49].

Anatomage utility in research

The Anatomage table offers research opportunities that could be used in the research of morphological parameters with the possibility of its application in anthropological research. In China, the morphometry of the temporomandibular joint was researched using the Anatomage table. The results proved the presence of a wide space of inter-fossa and long inter-condyle distance with the presence of asymmetries between two sides of mandibular condyles [50].

Another morphometric analysis research was done by using Anatomage for the detection of bone resection in anterior petrosectomy. Researchers removed tissue layers in order to demonstrate surgical procedures easier for junior surgeons [14].

Anatomage utility during the COVID-19 pandemic

Teaching and learning outcomes were markedly affected all over the world by coronavirus disease 2019 (COVID-19). Medical students get anatomical courses online through their platforms. The Anatomage table could represent a valuable resource to learn the student’s gross anatomy. Anatomage facilitated the surgical training program during COVID-19 since it can be uploaded with a software program for surgery [39]. Anatomage can also be used to gain 3D images of the lung that proved to be reliable with high accuracy for a COVID-19 diagnosis. Hasni and his coworkers demonstrated that in patients suffering from COVID-19, it is possible to diagnose COVID-19 by 3D reconstruction of CT scans and even the stage of disease for the suggestion of the appropriate protocol [29]. Anatomage could also be used for remote consultations.

## Conclusions

In conclusion, the present review article gives some insight into the benefits of the Anatomage virtual table and its utilities in education and diagnosis. Anatomical knowledge retention can be achieved by utilizing Anatomage in conjunction with cadaveric dissection. Further, it is an important tool for understanding congenital anomalies, anatomical structure variations, and normal and pathological cases. It is necessary for the training programs of students and residents and for disease diagnosis and prognosis. Introducing Anatomage into the curriculum can make undergraduate and postgraduate programs more interesting and valuable. Anatomage can also help in the assessment of students, physicians, and surgeons. Anatomage improved the learning outcome of anatomy, radiology, and surgery. At last, it has a crucial role during exceptional circumstances such as the pandemic crisis.
